# Long Non-Coding RNAs in Lung Cancer: The Role in Tumor Microenvironment

**DOI:** 10.3389/fcell.2021.795874

**Published:** 2022-01-03

**Authors:** Shuang Dai, Ting Liu, Yan-Yang Liu, Yingying He, Tao Liu, Zihan Xu, Zhi-Wu Wang, Feng Luo

**Affiliations:** ^1^ Department of Medical Oncology, Lung Cancer Center, West China Hospital, Sichuan University, Chengdu, China; ^2^ Department of Biotherapy, Cancer Center, West China Hospital, Sichuan University, Chengdu, China; ^3^ Oncology Department, People’s Hospital of Deyang City, Deyang, China; ^4^ Department of Oncology, The First Affiliated Hospital of Chengdu Medical College, Chengdu Medical College, Chengdu, China; ^5^ Department of Chemoradiotherapy, Tangshan People’s Hospital, Tangshan, China

**Keywords:** lncRNA, non-small cell lung cancer, immunotherapy, tumor microenvironment, biomaker

## Abstract

The development of various therapeutic interventions, particularly immune checkpoint inhibitor therapy, have effectively induced tumor remission for patients with advanced lung cancer. However, few cancer patients can obtain significant and long-lasting therapeutic effects for the limitation of immunological nonresponse and resistance. For this case, it’s urgent to identify new biomarkers and develop therapeutic targets for future immunotherapy. Over the past decades, tumor microenvironment (TME)-related long non-coding RNAs (lncRNAs) have gradually become well known to us. A large number of existing studies have indicated that TME-related lncRNAs are one of the major factors to realize precise diagnosis and treatment of lung cancer. Herein, this paper discusses the roles of lncRNAs in TME, and the potential application of lncRNAs as biomarkers or therapeutic targets for immunotherapy in lung cancer.

## Introduction

Lung cancer ranks the most important leading cause of cancer-related deaths globally (Bray et al., 2018; Nasim et al., 2019). Non-small cell lung cancer (NSCLC) accounts for more than 80% of all lung cancers (Bender, 2014). Despite the improvements of NSCLC treatment in traditional therapies, the overall cure and survival rates for NSCLC remain low, particularly in metastatic diseases (Siegel et al., 2020). Existing research suggests that combination treatment options (using immunotherapies or targeted therapies) may be the ultimate curative option. Hence, it is quite essential to investigate the precise molecular mechanism and biomarkers to promote the effectiveness of treatment, especially immune checkpoint inhibitors (ICIs).

ICIs reactivate dysfunctional and/or exhausted T cells by targeting immune checkpoints including cytotoxic T lymphocyte related protein 4 (CLTA-4), programmed cell death protein 1 (PD-1), or its ligand, PD-L1. To date, ICIs have altered treatment paradigm in multiple indications such as melanoma, NSCLC, renal cell carcinoma (RCC) and so on (Pardoll, 2012; Hoos, 2016; Papaioannou et al., 2016; Munn and Jain, 2019; Xin Yu et al., 2019). However, the treatment response of ICIs remains unsatisfactory, and the objective response rate (ORR) for ICIs alone is only about 15–25%, and even lower in pancreatic carcinoma, triple negative breast cancer, and colorectal cancer with microsatellite stability (MSS) (Gao et al., 2019). Most patients still face the dilemmas of primary/acquired resistance of ICIs. A great number of studies have investigated the resistance mechanisms that limit the efficacy of ICIs such as disability of neoantigen presentation, activation of T cell, the impaired formation of T cell memory and the dysregulation of tumor microenvironment (TME) (Gajewski et al., 2013; Hegde et al., 2016; Jenkins et al., 2018; Yi et al., 2019; Schoenfeld and Hellmann, 2020). TME, comprised of the interaction between tumor cells, tumor-associated stromal cells as well as extracellular matrix, has been considered to be of great significance in activating the effect of ICIs. Notably, identifying abnormal TME and treatment-related biomarkers are not only the important means of antitumor therapy, but also of immune efficiency improvement (Gao et al., 2019).

LncRNAs, the most frequently expressed nonprotein-coding RNAs, have at least 200 nucleotides and are usually located in the cell nucleus, cytoplasm and exosomes where they interact with various molecules like DNA, RNA, proteins and so forth (Atianand and Fitzgerald, 2014). Multiple pathophysiological processes through the epigenetic, transcriptional and post-transcriptional regulation of gene are regulated by lncRNAs, of which lncRNAs include at least five categories including intergenic lncRNAs, intronic lncRNAs, antisense lncRNAs, sense lncRNAs and bidirectional lncRNAs (pseudogenes and retrotransposons) (Rinn and Chang, 2012). In TME, lncRNAs can directly or indirectly affect the growth of tumor cells, and play a nonnegligible role in the regulatory recircuit of the immune cells, promoting recruitment of immunosuppressive cells such as Tregs, M2-type macrophages and myeloid-derived suppressor cells (MDSCs), down-regulating the expression of adhesion molecules on endothelial cells, as well as up-regulating of immune checkpoints (PD-1/PD-L1 and CTLA4), which could contribute to tumor development and resistance to drugs or radiotherapy (Fu et al., 2021; Taheri et al., 2021). Specifically, lncRNAs are new emerging therapeutic targets and important prognostic biomarkers in multiple cancers including lung cancer (Tokgun et al., 2020). Accumulating studies have identified that lncRNAs are essential mediators of intercellular communication between tumor and stromal cells in local and distant microenvironment of lung cancer.

**FIGURE 1:**
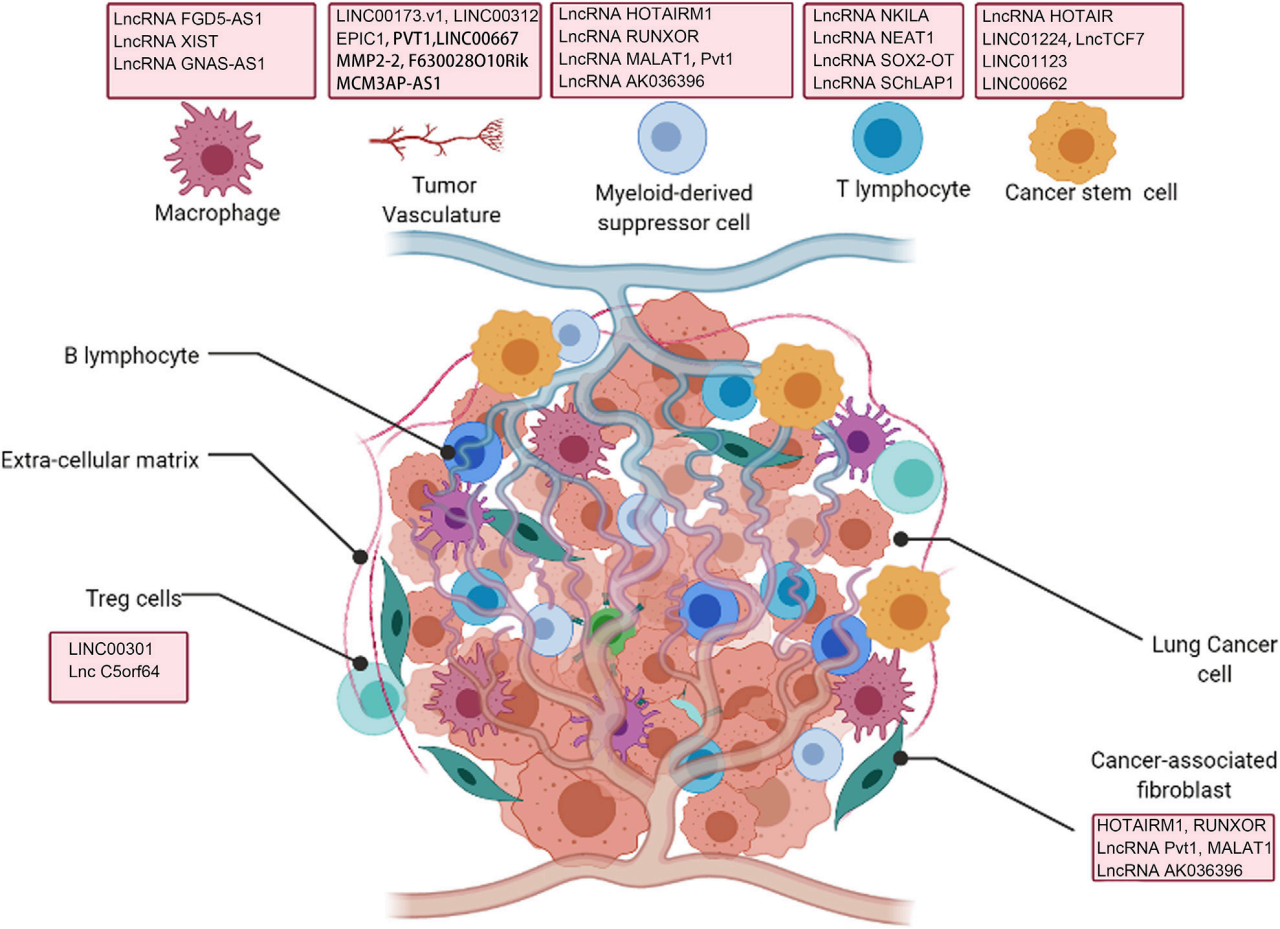
Interaction of lncRNAs with tumor microenvironment (TME) regulating lung cancer development.

In this review, we focus on describing how lncRNAs derived from tumor cells, immune cells or exosomes regulate the TME in lung cancer to promote tumor progression, emphasizing the role of these lncRNAs in tumor cells, lymphoid immune cells, macrophages, cancer-related fibroblasts, tumor vasculature and other components of TME (Figure 1).

## LncRNAs Affect Lymphoid Immune Cells

Existing research has reported that lymphoid immune cells within tumors have two-sided (positive and negative) effects on tumorigenesis and tumor progression (Joyce and Pollard, 2009). LncRNAs as important regulator molecules influence the activity and sensitivity of tumor-infiltrating T cells. Duan et al. conducted lncRNA profiling to screen for differentially expressed lncRNAs related to CD8^+^ T cells and activated memory CD4^+^ T cells in NSCLC (Duan et al., 2020). A total of 90 DElncRNAs (differential expression lncRNA) showed different expression patterns in CD8^+^ T immune cells, and 48 DElncRNAs were associated with activated memory CD4^+^ T cells. The enrichment pathway analyses revealed that differentially expressed lncRNAs were mainly involved in cytokine–cytokine receptor interaction as well as viral protein interaction with cytokine–cytokine receptor, suggesting that T cell-specific lncRNAs mediated an immune response during NSCLC progression. This study provided a cursory but comprehensive indication of the vital role of lncRNAs in influencing the biological function of T lymphoid cells in lung cancer. Specifically, a study reported that a large proportion of CTLs/Th1 cells underwent apoptosis in NSCLC, a process that could be further facilitated by anti-CD3, while the proportion of Th2 cells/Treg cells were relatively low in TME, suggesting CTLs/Th1 cells were more sensitive to activation-induced cell death (AICD) than Tregs/Th2 cells. Further analysis indicted that STAT1-mediated transcription of NKILA caused AICD of CTLs/Th1 cells by suppressing NF-κB which was upregulated in Tregs/Th2 cells compared with CTLs/Th1 cells (Huang et al., 2018). Tang et al. also observed that the propotion of Th2 was high in peripheral blood of NSCLC, and the proportion of CTLs/Th1 cell was lower than that in normal control. LncRNA NEAT1 was also reported to be upregulated in lung cancer tissues with high TILs. NEAT1 negatively regulated CD8^+^ T cells in lung cancer cells via increasing CXCL10, CCL5, and IFN-β expression, which directly induced cGAS/STING Signaling, to suppress immune response (Ma et al., 2020). In addition, signaling from SOX2-OT/miR-30d-5p/PDK1 contributed to apoptosis of CD8^+^ T cells, which in turn promoted immune escape of NSCLC (Chen et al., 2021). The direct interaction between SChLAP1 and AUF1 antagonized the binding between AUF1 and PD-L1 mRNA 3′-UTR, resulting in improving PD-L1 mRNA stability and expression, thereby attenuating CTLs function (Du et al., 2021). Collectively, lncRNAs control lung cancer development and metastasis by affecting T cell function in TME, and targeting lncRNA may alter the activity of CTLs, thereby enhancing the effectiveness of immunotherapy.

## LncRNAs in Cancer Stem Cells

Cancer stem cells (CSCs) are regarded as a population of tumor cells characterized by abilities to self-renew or to differentiate into cancer non-stem progenies, and therefore often involved in the resistance to cancer therapies, tumorigenesis, epithelial-to-mesenchymal transition (EMT) and tumor metastases (Clarke and Fuller, 2006; Zhao, 2016). CD44, CD133, OCT-4, Bmi-1, ALDH1, ABCG2 and KLF4 are common CSC biomarkers that are specifically and highly expressed on the cell surface. For example, Prior researches elucidated the determinants about the resistance to cancer treatments including growth features associated with slow division and quiescence (Vidal et al., 2014; Ajani et al., 2015), ATP-binding cassette (ABC) transporters expression levels involved in elimination of drugs (Abdullah and Chow, 2013), and the presence of increased detoxification of endogenous and exogenous aldehyde substrates via the aid of aldehyde dehydrogenases (ALDHs) (Sládek, 2003; Ahmed Laskar and Younus, 2019). As a pivotal component of the TME, the maintenance of CSCs as well as the growth and progression of tumors are inseparable from the cancer microenvironment, together with various regulatory factors. To date, lncRNAs involved in both CSCs biological functions and cancer development have received considerable research attention (Schwerdtfeger et al., 2021). Here we have reviewed relevant literatures to summarize the functions.

In regulating tumorigenesis, lncRNA HOTAIR was reported to exert pro-cancer effects by inducing CSCs and EMT formation under the direct regulation of STAT3 under cigarette smoke exposure (Liu et al., 2015). The relationship between stemness and EMT programs has been reviewed in previous literature (Wilson et al., 2020). DUXAP10 was obviously upregulated in Cd-induced lung cancer cells. DUXAP10 knockdown induced the stemness markers including KLF4, KLF5 and Nanog downregulation, weakened the capacity of spheres formation and reduced the number of stem cells marked by CD133 via inhibiting the Hedgehog signaling pathway signal, which was involved in Cd carcinogenesis in lung cells (Lin et al., 2021). As for resistance to cancer therapies, Liu et al. revealed that lncRNA HOTAIR could cause cisplatin resistance via inducing stem cell-related biomarkers β-catenin and KLF4, especially directly regulating KLF4, to promote stemness (Liu et al., 2016). Similarly, LINC01224 is obviously upregulated in NSCLC cells and associated with NSCLC radioresistance. LINC01224 knock-down dramatically promotes the abilities of self-renew by regulating the expression of ZNF91 and therefore suppresses the irradiation sensitivity of NSCLC (Fu et al., 2021). In terms of EMT and metastasis, FOXF1-AS1 acted as a protective factor and interacted with PRC2 components EZH2 to hinder self-renewal of NSCLC CSCs and reduce the number of stem-like cells, thus leading to impaired EMT capacity of tumor cells (Miao et al., 2016). LncTCF7 overexpression increased NSCLC sphere formation and expression of specific markers (EpCAM, Sox2, Oct4 and Nanog). Further study of the molecular mechanism of TCF7 regulation of CSC revealed that TCF7 may exhibit oncogenic activity by regulating EpCAM via competitively binding miR-200c, which stimulated invasive activity and enhanced the self-renewal capacity of CSCs (Wu and Wang, 2017). The interaction with LINC00662 and its RNA binding protein Lin28 can elevate CSCs stemness and invasion ability of tumor cells, contributing to the poor prognosis of NSCLC (Gong et al., 2018). The expression of LINC01123 is up-regulated in LUAD. Mechanistically, LINC01123 could precipitate miR-449b-5p to release NOTCH1, thereby promoting downstream NOTCH1 signaling and resulting in accelerating LUAD cell stemness and EMT (Zhang et al., 2020). More LncRNAs affecting CSC properties are summarized in Table 1. Taken together, all these data elucidate that dysregulation of lncRNAs affects CSCs traits and tumor invasion, and could be potential targets of tumor immunotherapy.

**TABLE 1:** LncRNAs and their respective molecules or pathways involved in theTME.

LncRNA	Effects	Mechanism	References
FGD5-AS1	M2	Regulating FGD5-AS1/miR-944/MACC1 axis	Lv et al. (2021)
XIST	M2	Regulates M2 polarization	Sun and Xu (2019)
GNAS-AS1	M2	GNAS-AS1/miR-4319/NECAB3 axis	Li et al. (2020a)
SOX2-OT	M2	Targeting miR-627-3p/Smads signaling pathway	Zhou et al. (2021)
NKILA	CTLs/Th1	Enhancing AICD of CTLs/Th1 cells by suppressing NF-κB	Huang et al. (2018)
NEAT1	CTLs	Suppressing cGAS/STING Signaling	Ma et al. (2020)
SOX2-OT	CTLs	SOX2-OT/miR-30d-5p/PDK1	Chen et al. (2021)
SChLAP1	CTLs	Regulating the AUF1/PDL1 axis	Du et al. (2021)
HOTAIR	CSCs	Inducing EMT and CSCs under the direct regulation of STAT3	Liu et al. (2015)
HOTAIR	CSCs	Inducing CSCs-related biomarkers β-catenin and Klf4	Liu et al. (2016)
FOXF1-AS1	CSCs	Interacting with EZH2 to inhibit the EMT ability of tumor cells	Miao et al. (2016)
TCF7	CSCs	Regulating TCF7/miR-200c/EpCAM	Wu and Wang (2017)
LINC00662	CSCs	Interacting with Lin28	Gong et al. (2018)
DUXAP10	CSCs	Inhibiting the Hedgehog signaling pathway signal	Lin et al. (2021)
MCF2L-AS1	CSCs	Regulating miR-873-5p	Li and Lin (2021)
LINC01224	CSCs	Interacting with ZNF91 dove irradiation resistance	Fu et al. (2021)
LINC01123	CSCs	Precipitating miR-449b-5p to activate NOTCH1 pathway signal	Zhang et al. (2020)
DANCR	CSCs	Activating DANCR/miR-216a signaling axis	Yu et al. (2020)
DHRS4-AS1	CSCs	Modulating DHRS4-AS1/miR-224-3p signaling	Yan et al. (2020)
MACC1-AS1	CSCs	MACC1-AS1/UPF1/LATS1/2 axis	Wang et al. (2020)
loc107985872	CSCs	Activating the notch1 signaling pathway	Guo et al. (2020)
SLNCR1	CSCs	Interacting with sPLA2	Xu et al. (2019)
LINC00887	CSCs	Stimulating multiple microRNAs (miRNAs)	Tian et al. (2019)
HAND2-AS1	CSCs	Interacting negatively with TGF-β1	Miao et al. (2019)
TUSC-7	CSCs	sponging miR-146	Huang et al. (2019)
CASC11	CSCs	Interacting with TGF-β1 to increase stemness of CSCs	Fu et al. (2019)
CCAT1	CSCs	Activating Wnt signalling	Xu et al. (2018)
DGCR5	CSCs	DGCR5/miR-330-5p/CD44 axis	Wang et al. (2018)
NEAT1	CSCs	Activating Wnt signalling	Jiang et al. (2018)
HOTAIRM1	MDSCs	Targeting HOXA1	Tian et al. (2018a)
RUNXOR	MDSCs	Regulating RUNX1 mRNA	Tian et al. (2018b)
LncRNA Pvt1	MDSCs	Attenuating Arg1 activity and ROS production	Zheng et al. (2019)
AK036396	MDSCs	Repressing Arg1 activity *in vitro* and CD244 expression	Tian et al. (2020)
MALAT1	MDSCs	Unknown	Zhou et al. (2018)
LINC00301	Tregs	Accumulating Tregs upon targeting TGF-β1	Sun et al. (2020)
C5orf64	Tregs	Decreasing Tregs abundance	Pang et al. (2021)
NRK	CAFs	Unknown	Wei et al. (2021)
LINC00173.v1	Vasculature	Sponging miR-511-5p as a ceRNA	Chen et al. (2020)
EPIC1	Vasculature	Ang2 -Tie2 signaling pathway	Hou et al. (2021)
LINC00667	Vasculature	Inducing eukaryotic translation initiation factor 4A3 (EIF4A3)	Yang et al. (2020)
F630028O10Rik	Vasculature	Sponging miR-223-3p	Qin et al. (2020)
MCM3AP-AS1	Vasculature	Targeting miR-340-5p/KPNA4 axis	Li et al. (2020b)
PVT1	Vasculature	Targeting the miR-29c/VEGF signaling pathway	Mao et al. (2019)
LINC00312	Vasculature	Binding YBX1	Peng et al. (2018)
lnc-MMP2-2	Vasculature	Regulating MMP2 expression	Wu et al. (2018)

LncRNAs: Long Non-coding RNAs; CSCs: Cancer stem cells; MDSCs: Myeloid-derived suppressor cells; Tregs: Regulatory T cells; M2: alternatively activated macrophage.

## LncRNAs Contribute to Macrophage Plasticity

Macrophages, most of which derived from blood monocytes, are involved in EMT and are present in almost all tissues such as hepatic Kupffer cells (KCs) or brain microglia (Varol et al., 2015). According to the different mechanisms of action, macrophages are roughly divided into classically activated macrophages known as “killer” macrophages (M1) activated by IFN-γ, TNF-α as well as lipopolysaccharide (LPS) and alternatively activated macrophages known as “repair” macrophage (M2) activated by IL-4, IL-10 or IL-13 (Li et al., 2019). Basic studies have indicated that in mouse models, high levels of MHC II molecules are expressed in tumor-associated macrophages (TAMs) with the hallmark of M1 during the early stages of tumor development. However, the advanced stage of tumor is mainly characterized by low-level MHC II molecules of M2, which indicates that an M1-to-M2 transformation is present in tumor progress (Mantovani et al., 2002; Wang et al., 2011). Obviously, the accumulation of macrophages is related to tumor angiogenesis, tumor invasion and immunosuppression (Condeelis and Pollard, 2006). LncRNAs expression has been implicated in many cellular and developmental processes like cell proliferation and apoptosis (Batista and Chang, 2013; Heward and Lindsay, 2014). Prior reports have suggested that lncRNAs are also involved in regulating macrophage polarization (Huang et al., 2016).

In recent years, it has been shown that lncRNAs originating from tumor cells are involved in the polarization of TAMs and result in tumor progression. Tumor cell-derived FGD5-AS1 *via* exosomes transportation promotes upregulation of M2 polarization markers (CD163, CD206, ARG1) and downregulation of M1 macrophage markers iNOS and IL-2 in NSCLC (Lv et al., 2021). Sun et al. summarized that the upregulated lncRNA XIST promoted macrophage conversion to M2 characterized by the deletion of specific makers like IL-10 and CD163, to affect tumor invasion and migration of lung cancer (Sun and Xu, 2019). GNAS-AS1 inhibits miR-4319 expression, and consequently activates N-terminal EF-hand calcium binding protein 3 (NECAB3) in THP-1-differentiated macrophages. Therefore, GNAS-AS1 increases the number of M2 macrophage and consequently promoting NSCLC cell growth and metastasis (Li Z. et al., 2020). Moreover, tumor-derived exosomal SOX2 overlapping transcript also play an important role in modulating the polarization of TAMs in NSCLC though regulating SOX2/miR-627-3p/Smads axis (Zhou et al., 2021). Taken together, lncRNAs directly or indirectly regulate the polarization of TAMs to affect lung cancer progression and metastasis, but the specific regulatory mechanism controlling the macrophages M2 polarization in lung cancer needs to be further studied.

## LncRNAs Promote by Regulating Immune Suppressive Cells

Studies have shown that the interactions between non-tumor cells exposed to the tumor microenvironment and tumor cells contribute to tumor progression and metastasis. In addition, T cells in tumors are often dysregulated and unable to generate specific responses to tumor cells in a timely manner. Immunosuppressive cells, including regulatory T cells (Tregs) usually expressing CD4, CD25 and FOXP3 markers (T cells that are not immunosuppressive) and MDSC, can contribute directly or indirectly to immunosuppression, which are reviewed in detail below.

### Myeloid-Derived Suppressor Cells

Myeloid-derived suppressor cells (MDSCs) express two specifical markers, CD11b and Gr1, and represent a heterogeneous population of myeloid origin that are activated and proliferated by growth factors and cytokines released by tumor cells. Once MDSCs are activated, they accumulate in lymphoid organs and tumors and exert immunosuppression on T cells. Currently, the cellular mechanisms by which MDSCs have been shown to be involved in immunosuppressive activity are: 1) inhibition of CTL cells activation and proliferation in an MHC-restricted or unrestricted and antigen-specific manner (Nagaraj et al., 2007; Zou et al., 2021); 2) indirectly affects T cell activation via the induction of Treg proliferation and benefiting from TGF, IL-10 production (Serafini et al., 2008); 3) Stimulation of macrophage conversion to M2 by secreting IL-10 and down-regulation of IL-12 that promotes M1 generation (Sinha et al., 2007); 4) interaction with type II iNKT, which promotes tumor progression through IL-13 production, thereby inducing aggregation of MDSCs (Zou et al., 2021). Of note, MDSCs have immunosuppressive activity only when activated. The molecules that can activate MDSCs include two main categories: 1) tumor-derived soluble factor (TDSF), which inducing the proliferation of MDSCs though activating STAT3 to stimulate the proliferation of myeloid cells and inhibit the differentiation of mature myeloid cells, including VEGF, SCF, GM-CSF, G-CSF, IL-6, IL-10, IL-12, MMP9 and CCL2 (Talmadge, 2007; Marigo et al., 2008); 2) soluble factors released by activated T cells and tumor-derived stromal cells, such as IFN-γ, TLRs ligands, IL-4, IL-13 as well as TGF-β, are responsible for the activation of different transcription factors such as STAT6, STAT1 and NFγB (Gabrilovich and Nagaraj, 2009). Recently, it has been shown that lncRNAs affect lung cancer progression by regulating the immunosuppressive function of MDSCs in TME. Tian et al. discovered that HOTAIRM1 is obviously downregulated in MDSCs and its expression reduces in peripheral blood of lung cancer. Overexpression of HOTAIRM1 can positively target HOXA1 and induce subsequent reduction of the immunosuppression function of MDSCs, as well as increase the number of Th1/CD8+ cytotoxic T lymphocyte cells (CTLs), thereby sustaining improving the antitumor immune response (Tian et al., 2018a). Moreover, they also observed that the expression of lncRNA RUNXOR is higher in the blood of lung cancer patients than the levels in healthy samples, while decreases after surgery. Further detection suggested that RUNXOR can promote the activation of MDSCs and decrease the proportion of Th1/CTL cells by regulating RUNX1 mRNA expression (Tian et al., 2018b). A study on lncRNA MALAT1 indicated that MALAT1 directly affected MDSCs differentiation in lung cancer (Zhou et al., 2018). Some studies are more detailed. Zheng et al. identified that the expression level of lncRNA Pvt1 was upregulated in G-MDSCs following induction of IL-6 and GM-CSF. In contrast, when Pvt1 was knocked down, Arg1 activity and ROS production were significantly reduced and the ability of G-MDSCs to suppress T cells turned weak. In mice injected with Lewis lung carcinoma cells, they found that the number of CTLs/Th1 cells increased compared with the normal treatment group. The function of G-MDSCs was obviously upregulated under hypoxic conditions though targeting HIF-1α, and inhibition of HIF-1α by YC-1 apparently reduced Pvt1 expression in G-MDSCs (Zheng et al., 2019). Owing to the diverse phenotype of MDSCs, MDSCs tend to be classified into CD11b^+^Ly6G^+^Ly6C^low^ polymorphonuclear MDSCs (PMN-MDSCs) and CD11b^+^Ly6G^+^Ly6C^hi^ monocytic MDSCs (M-MDSCs) in mice (Youn et al., 2008). A latest study revealed that LncRNA AK036396 had a fairly high level of expression in PMN-MDSCs, whereas knockdown of AK036396 repressed Arg1 activity *in vitro* and CD244 expression leading to reduction of immunosuppressive effects of PMN-MDSCs (Sagiv et al., 2015). At the mechanistic level, the researchers found that lncRNA AK036396 could interact with Fcnb through abrogating its ubiquitination to enhance its stability in the cytoplasm of myeloid cells, to enhance the immunosuppression of PMN-MDSCs and attenuate Th1/CTL cells responses, and ultimately accelerating tumor progression (Tian et al., 2020). These studies have demonstrated that lncRNAs play a significant role in the aggregation and activation of MDSCs. However, the role and regulatory mechanism during tumor progression of these lncRNAs within MDSCs in lung cancer awaits further exploration.

### Treg Cells

Regulatory T cells (Tregs), as a major subset of infiltrating CD4^+^ T cells in TME, specifically express the master transcription factor FOXP3 (Sakaguchi et al., 2020), and have been found to suppress anti-tumor immune responses in diverse ways, including: 1) targeting TGF-β, thus inhibits the anti-tumor effects promoted by CD4^+^ cells, CD8^+^ cells, and NK cells (Konkel and Chen, 2011; Sun et al., 2020); 2) disrupting metabolism by scavenging cytokines such as IL-2, or producing immunosuppressive adenshakes by extracellular enzymes CD39 and CD73 (Spolski et al., 2018); 3) inhibiting the maturation and function of DC; 4) dissolved by granulase A or B and perforation induced CD8^+^ lymphocytes. Several studies involving humans and mice have shown that extrinsic tissue and tumors in different tissues contain the largest Tregs, and that the absence of Treg cells can significantly improve anti-tumor immunity. A few studies reported that lncRNA affects tumor progression by regulating the biological behaviors and function of Tregs. Sun et al. identified a novel lncRNA, LINC00301, that can promote the accumulation of Tregs and decrease CD8^+^ T cell in NSCLC upon targeting TGF-β1 (Sun et al., 2020). Moreover, lncRNA C5orf64 expression is positively correlated with NSCLC survival, but negatively associated with Tregs levels (Pang et al., 2021).

## The Role of LncRNA in Cancer-Associated Fibroblasts

Cancer-associated fibroblasts (CAFs), as one of major components in TME that derive from the differentiation of quiescent fibroblasts by activation of various external factors like cytokines/chemokines, growth factors, hypoxia factors, lncRNAs and so on, participate in the entire cancer developmental process, from tumor initiation to progression, including carcinogenesis, proliferation, migration, EMT, drug resistance, metabolic reprogramming, angiogenesis and immunosuppression (Wu et al., 2017; Shoucair et al., 2020; Yang et al., 2021). Growing studies also reveal that lncRNAs also play a nonnegligible role in cancer cells and CAFs by shuttling via exosomes and directly within CAFs or cancer cells. But, the roles of lncRNAs in CAFs during lung cancer progression indeed remain unclear and are poorly studied. Teng et al. had attempted to identify differentially expressed lncRNAs between CAFs and normal fibroblasts in NSCLC using lncRNA profiling analysis with the intention of selecting important biomarkers working in TME. They found that upregulated lncRNAs were involved in important cancer-related regulatory pathways such as NOD-like receptor signaling (Teng et al., 2019). The roles and mechanisms of action of individual lncRNAs in CAFs of lung cancer are starting to be realized.

## The Tumor Vasculature is Supported by LncRNAs

In physiological environment, angiogenesis maintains a relatively dynamic homeostasis and is strictly controlled by pro-angiogenesis and anti-angiogenesis regulators (Ribatti et al., 2007). Hypoxia and acidosis in tumor bed are often attributed to a large consumption of oxygen and nutrients and an active metabolism under a disproportionate blood supply (Kerbel, 2008; Sun, 2012). In this case, it continues to induce the production of large amounts of pro-angiogenic factors in TME (Ronca et al., 2017). Meanwhile, various angiogenic factors such as Vascular endothelial growth factor (VEGF) (Ferrara et al., 2003), angiopoietin (ANGPT) (Fagiani and Christofori, 2013) and basic fibroblast growth factor (bFGF) (Zheng et al., 2018) also increase and have immunosuppressive functions. As a result, the balance of pro- and anti-angiogenesis is disturbed in cancer, leading to a shift to angiogenesis and immunosuppressive microenvironment (Ribatti et al., 2007). Some evidence has shown that lncRNAs act on tumor progression by regulation of VEGF in lung cancer. A report (Chen et al., 2020) showed that LINC00173.v1 is upregulated in lung squamous cell carcinoma (SQC) tissues and is negatively associated with SQC prognosis. Knockdown of LINC00173.v1 suppresses VEGFA expression, thus attenuating vascular endothelial cell proliferation and migration, as well as tumorigenesis of SQC cells. Mechanistic evidence has exhibited that LINC00173. v1 exerts these functions by sponging miR-511-5p as a ceRNA. Hou et al. (2021) reported that lncRNA EPIC1 is significantly upregulated in NSCLC tissues and cells. EPIC1 silence represses Ang2 -Tie2 signaling pathway-related proteins, thereby inhibiting HUVECs (human umbilical vein endothelial cell) proliferation and channel forming abilities. lncRNA LINC00667 induces eukaryotic translation initiation factor 4A3 (EIF4A3) expression and secretion, and consequently activates the mRNA and protein levels of VEGFA. Therefore, LINC00667 promotes angiogenesis of NSCLC cells and consequently results in NSCLC tumor growth and metastasis (Yang et al., 2020). Knockdown of lncRNA F630028O10Rik in lung cancer increases VEGFA and VEGFR2 expression by sponging miR-223-3p, which means that F630028O10Rik could inhibit tube formation in vascular endothelial cells, thus further influencing angiogenesis in lung cancer (Qin et al., 2020). Besides, lncRNAs, MCM3AP-AS1 (Li X. et al., 2020) and PVT1 (Mao et al., 2019) also play a key role in accelerating angiogenesis in lung cancer, respectively. These researches provide a rationale for using the anti-angiogenic effects of lncRNAs as a therapeutic option for lung cancer. Yet, the effect on tumor angiogenesis for lncRNAs still awaits further investigation.

## LncRNAs as Prognostic Biomarkers

**FIGURE 2:**
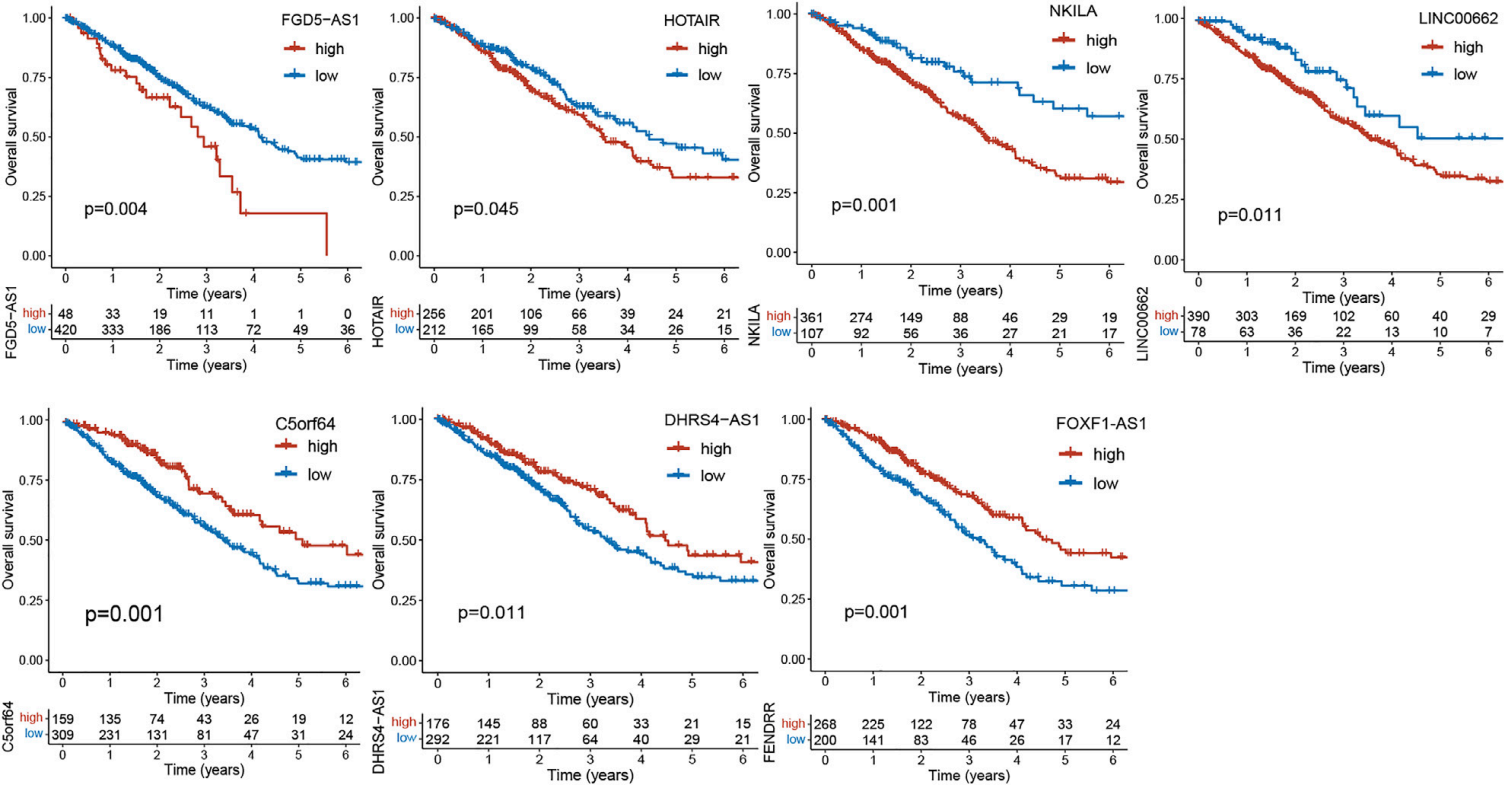
Kaplan-Meier curves of overall survival (OS) between low- and high-expression groups of lncRNAs in the TCGA cohort.

Diagnosis and therapies using lncRNAs are being developed. For example, the investigators have conducted clinical settings to identify lncRNA biomarkers from the plasma to facilitate detection of early lung cancer (NCT03830619). Studies have suggested that lncRNAs not only interact with the TME, but closely correlate with cancer prognosis. We extracted a number of gene expression profiles of lncRNAs in Lung adenocarcinoma from the TCGA database (*N* = 468). Based on the best cutoff value of gene expressions, we performed Kaplan-Meier survival analysis to validate the association between part of lncRNAs mentioned above and prognosis of lung cancer using the *R* package “Survminer”. As illustrated in Figure 2, the key lncRNAs exhibit good performance in prognostic prediction of lung cancer (*p* < 0.05). For instance, FDG5-AS1, HOTAIR, NKILA and LINC00662 are regarded as risk factors for survival of lung cancer (Liu et al., 2015; Gong et al., 2018; Huang et al., 2018; Lv et al., 2021), while C5orf64, DHRS4-AS1 and FOXF1-AS1 are protective factors. These genes have important effects on lung cancer (Miao et al., 2016; Yan et al., 2020; Pang et al., 2021). As far as these lncRNAs are concerned, although there is no specific clinical application in lung cancer to date, they are still potential for the comprehensive treatment and diagnosis of lung cancer.

## Conclusion and Future Perspectives

Lung cancer is a highly malignant tumor that poses a serious threat to human health and life. There is a lack of effective means to identify early-stage lung cancer, leading to high mortality and failure of comprehensive interventions. Meanwhile, with advances in immunotherapy, the role of TME in the lung cancer diagnosis and prognostic is becoming increasingly critical. Components of TME interacting with lncRNA have been shown to contribute to immunomodulation and cancer progress. Hence, we summarized recent advancement involving lncRNAs and their roles in the crosstalk between components of TME including infiltrated immune cells, CSCs, immune suppressive cells, macrophage, CAFs and part of the underlying molecular mechanisms. So far, only a small number of lncRNAs have been well elucidated in tumor-mesenchymal crosstalk, and in-depth studies are worthwhile to identify more lncRNAs and their specific biological functions and mechanisms of involvement. A deeper understanding of the role played by lncRNAs in the tumor microenvironment may greatly facilitate further discovery of potential biomarkers and the development of novel targeted therapies for the treatment of lung cancer. To date, the pressing issue has been the in-depth and systematic elucidation of the regulatory determinant mechanism of lncRNAs.

In conclusion, the important regulatory roles of lncRNAs in the TME have been gradually described; however, the clinical applications of lncRNAs still need to be further explored. Along with further research, tumor-associated lncRNAs crosstalk will open a new era of anti-tumor therapy.

## Abbreviations

LncRNAs, Long Non-coding RNAs; LUAD, Lung adenocarcinoma; TME, Tumor microenvironment; ICIs, Immune checkpoint inhibitors; NSCLC, Non-small cell lung cancer; SQC, Lung squamous cell carcinoma; CLTA-4, cytotoxic T lymphocyte related protein 4; ORR, objective response rate; M1, Classically activated macrophages; M2, alternatively activated macrophage; MPS, monocyte macrophage system; EMT, Epithelial-to-mesenchymal transition; KCs, Kupffer cells; CTLs, Cytotoxic T lymphocyte cells; VEGF, Vascular endothelial growth factor; CSCs, Cancer stem cells; MDSCs, Myeloid-derived suppressor cells; Tregs, Regulatory T cells; VEGFA: vascular endothelial growth factor A.
